# Burden of oral anticoagulation in embolic stroke of undetermined source without atrial fibrillation

**DOI:** 10.1186/s12872-021-01967-x

**Published:** 2021-03-31

**Authors:** Klaus K. Witte, Georgios Tsivgoulis, Matthew R. Reynolds, Stelios I. Tsintzos, Simon Eggington, Eleni Ismyrloglou, Julie Lyon, Marianne Huynh, Marta Egea, Bonnie de Brouwer, Paul D. Ziegler, Noreli Franco, Rashmi Joglekar, Sarah C. Rosemas, Shufeng Liu, Vincent Thijs

**Affiliations:** 1 Leeds Institute of Cardiovascular and Metabolic Medicine, University of Leeds, LIGHT Building, Clarendon Way, Leeds, LS2 9JT UK; 2 Second Department of Neurology, “Attikon” University Hospital, National & Kapodistrian University of Athens Medical School, Athens, Greece; 3 Baim Institute for Clinical Research, Boston, MA USA; 4 Medtronic International Trading Sarl, Tolochenaz, Switzerland; 5 Medtronic Bakken Research Center B.V., Maastricht, Netherlands; 6 Medtronic UK and Ireland, Watford, UK; 7 Medtronic Australasia Pty Ltd, North Ryde, Australia; 8 Medtronic Spain, Madrid, Spain; 9 Medtronic Global CRHF Headquarters, Mounds View, MN USA; 10 Florey Institute of Neuroscience, Melbourne, Australia

## Abstract

**Objective:**

Prevention of recurrent stroke in patients with embolic stroke of undetermined source (ESUS) is challenging. The advent of safer anticoagulation in the form of direct oral anticoagulants (DOACs) has prompted exploration of prophylactic anticoagulation for all ESUS patients, rather than anticoagulating just those with documented atrial fibrillation (AF). However, recent trials have failed to demonstrate a clinical benefit, while observing increased bleeding. We modeled the economic impact of anticoagulating ESUS patients without documented AF across multiple geographies.

**Methods:**

CRYSTAL-AF trial data were used to assess ischaemic stroke event rates in ESUS patients confirmed AF-free after long-term monitoring. Anticipated bleeding event rates (including both minor and major bleeds) with aspirin, dabigatran 150 mg, and rivaroxaban 20 mg were sourced from published meta-analyses, whilst a 30% ischaemic stroke reduction for both DOACs was assumed. Cost data for clinical events and pharmaceuticals were collected from the local payer perspective.

**Results:**

Compared with aspirin, dabigatran and rivaroxaban resulted in 17.9 and 29.9 additional bleeding events per 100 patients over a patient’s lifetime, respectively. Despite incorporating into our model the proposed 30% reduction in ischaemic stroke risk, both DOACs were cost-additive over patient lifetime, as the costs of bleeding events and pharmaceuticals outweighed cost savings associated with the reduction in ischaemic strokes. DOACs added £5953–£7018 per patient (UK), €6683–€7368 (Netherlands), €4933–€9378 (Spain), AUD$5353–6539 (Australia) and $26,768–$32,259 (US) of payer cost depending on the agent prescribed. Additionally, in the U.S. patient pharmacy co-payments ranged from $2468–$12,844 depending on agent and patient plan. In all settings, cost-savings could not be demonstrated even when the modelling assumed 100% protection from recurrent ischaemic strokes, due to the very low underlying risk of recurrent ischaemic stroke in this population (1.27 per 100 patient-years).

**Conclusions:**

Anticoagulation of non-AF patients may cause excess bleeds and add substantial costs for uncertain benefits, suggesting a personalised approach to anticoagulation in ESUS patients.

**Supplementary Information:**

The online version contains supplementary material available at 10.1186/s12872-021-01967-x.

## Key messages

- In ESUS patients from CRYSTAL-AF confirmed as being free of AF with three years of continuous monitoring, the recurrent stroke rate is low such that any savings from reduced ischaemic stroke recurrence is likely to be offset by increases in bleeding-related and prescription costs.
- Our results demonstrate that DOACs would generally be cost-additive because of the very low rate of stroke and the additional risk of bleeding.
- In environments with substantial patient participation in hospitalisation and prescription expenses, DOACs in this population may add substantial out-of-pocket patient costs for uncertain benefits.

## Introduction

In routine practice, despite comprehensive evaluation, the aetiology of up to 40% of ischaemic strokes remains unknown. Initially labelled as cryptogenic strokes (CS) [1, 2], to reduce heterogeneity diagnostic criteria were refined with most CS cases now termed Embolic Strokes of Undetermined Source (ESUS), representing 32% of ischaemic stroke cases [3–5].

Intermittent and asymptomatic atrial fibrillation is an aetiological factor for ischaemic stroke. The CRYSTAL-AF trial [6] showed an AF detection rate of 30.0% after three years of continuous monitoring with an insertable cardiac monitor (ICM) in patients with CS, higher at 33.6–35.8% in those patients fulfilling ESUS criteria [7]. Guidelines remain clear on the need for AF patients with sufficient risk factors to be anticoagulated to reduce the risk of primary or recurrent stroke [8–10]. However, until recently, whether anticoagulation might be useful in CS (or ESUS) patients without proven AF was unknown. Randomised controlled clinical trials (RCTs) of mostly warfarin in patients without AF [11–13] had not confirmed a net benefit of anticoagulation in the absence of AF. Specifically, reductions in ischaemic strokes were counteracted by increases in bleeding, leaving aspirin unchallenged as the antithrombotic of choice in the absence of AF. Nevertheless, questions remained about whether safer anticoagulants could improve upon the therapeutic profile of aspirin for stroke prevention.

RCTs have shown that direct oral anticoagulants (DOACs) match or exceed warfarin in efficacy and safety for patients with AF [14–17], and several economic evaluations have determined DOACs to be cost-effective for the prevention of stroke in patients with documented AF, as compared to warfarin or aspirin [18, 19]. Their improved safety profile over warfarin led to the hypothesis that they were sufficiently safe to be prescribed in patients with risk factors, but without confirmed AF. Recently published trials using dabigatran and rivaroxaban designed to answer this question in the ESUS population reported no overall change in ischaemic stroke rate but increased bleeding [20, 21].

Considering these recent developments, we aimed firstly to provide a plausible prediction, and then explanation, for these results by better understanding the potential clinical impact of DOAC therapy with dabigatran and rivaroxaban in ESUS patients proven not to have AF, using the unique opportunity provided by the CRYSTAL-AF dataset. Secondly, we sought to describe the economic consequences across multiple international settings (the United States (U.S.), United Kingdom (UK), Spain, Netherlands, and Australia) of offering anticoagulation to all ESUS patients.

## Methods

The analysis first required data on event rates per patient per year for recurrent ischaemic stroke and major and minor bleeding events for the ESUS patient population. To project outcomes to patients’ lifetimes, and thus understand the consequences of anticoagulating the subgroup of patients with an ESUS but in whom AF has been excluded, we used an existing economic framework by Diamantopoulos et al. [22] based upon data from the CRYSTAL-AF trial of ICMs in a CS patient population.

### Clinical event rates

Recurrent stroke rates were assessed in ESUS patients in the CRYSTAL-AF trial [7] who met the enrollment criteria for both NAVIGATE ESUS [20] and RE-SPECT ESUS [21] studies, in whom no AF of any duration had been detected by the end of follow-up despite 36 months of continuous monitoring. We focused solely on recurrent stroke events and excluded transient ischaemic attacks (TIAs).

Bleeding event rates for dabigatran 150 mg and rivaroxaban 20 mg were sourced from the literature as described by the aforementioned economic framework [22], supplemented with data from a recent network meta-analysis by Tawfik et al. [23] which presented event rates in AF patients enrolled in 16 RCTs with warfarin and DOACs against aspirin. Major bleeds included in the model were haemorrhagic stroke and other intracranial haemorrhage, while minor bleeding included extracranial haemorrhages and clinically-relevant non-major (CRNM) [24] bleeds. To align with the powering assumptions used for the designs of the RESPECT ESUS and NAVIGATE ESUS trials, we assumed that these agents would result in a 30% relative risk reduction for ischaemic stroke against aspirin. Risks of stroke/bleed events and treatment effects were modeled over the lifetime of the patient, with risks adjusted by a factor of 1.46 and 1.97 per decade age for ischaemic strokes and bleeding events, respectively [20].

### Long-term clinical projection framework

The previously described Markov model for ICM use in CS [22] was adapted to be used as a cost-consequence analysis on medication and event costs over a patient lifetime. The aspirin arm of the model was used as the foundation. Baseline characteristics of the patients were equalized with the ESUS subgroup in the CRYSTAL-AF trial: mean age 59.6 years, 58.2% male, mean CHADS_2_ score 2.8. Incidence of AF was zeroed since we informed the model using patients free of AF. The model was then run separately for aspirin, rivaroxaban 20 mg, and dabigatran 150 mg using the DOAC-specific bleed rates from the literature and assumed ischaemic stroke rates in line with our assumptions as described above. For lack of better data, stroke severity information remained as in the original economic model. Patient survival after recurrent stroke was informed by adjusted survival curves for each of the healthcare environments studied. Details can be found in the Additional file 1: Supplementary Appendix. Discounting of costs and outcomes was applied in-line with the discounting guidelines of each healthcare setting (3–5% per annum). Due to analytical constraints the model was unable to calculate the effects of more than one recurrent stroke (either an ischaemic or haemorrhagic stroke). For the other event types, including Other ICH, ECH, or CRNM bleeds, patient risks were not limited to one event. The model assumed full adherence to prescribed medications unless a major bleed occurred, following which medications were considered to be stopped.

### Cost inputs

Cost data for clinical events and pharmaceuticals were collected from the local healthcare system perspective for each of the examined environments (U.S., UK, Spain, Netherlands and Australia), and were inflated to 2017 dollars.

For the U.S., medication costs were derived from calendar year 2015–2016 Medicare 100% data, using the Instant Health Data (IHD) software (Panalgo, Boston MA, U.S.) and R, version 3.2.1. (R Foundation for Statistical Computing, Vienna, Austria). For amounts paid for ischaemic stroke hospitalisations and bleeding events we used 100% Medicare sample data from 2015–2016. Any extreme outliers were adjusted for by excluding subjects whose costs exceed 1.96 times the standard deviation of the mean cost in each cost extraction, in order to trim data which could be caused by billing data entry errors in the administrative datasets. Long-term (post-90 days) costs for stroke rehabilitation were sourced from the literature [25].

For the UK, stroke hospitalization costs were sourced from the literature [26], 2016–2017 Healthcare Resource Group (HRG) tariffs were used for other bleed-related hospitalisation costs, and 2017 British National Formulary (BNF) data for pharmaceutical pricing. Spanish hospitalisation costs were informed by 2017 data of the Health Information Institute of the Ministry of Health and Social Services (Instituto de Información Sanitaria; Ministerio de Sanidad, Servicios Sociales e Igualdad) and long-term stroke costs from the literature [27]. Dutch hospitalisation costs were sourced from data of the Dutch Healthcare Authority (Nederlandse Zorgautoriteit—NZa) and medication costs were based on the Pharmacy Purchase Price as provided by the Netherlands Costing Institute (Zorginstituut Nederland), both from calendar year 2017. Australian costs were sourced from a recent health technology assessment (HTA) on ICMs in CS [28]. Further details are provided in the Additional file 1: Supplementary Appendix.

### Patient economic burden (United States)

We estimated the economic burden of the examined strategies to patients directly. We identified the level of preference for some of the top private payers currently offering plans in the U.S. (American Association of Retired Professionals “AARP”, United Healthcare, Anthem, Aetna) and in combination with IHD cost data estimated the level of co-pay for pharmaceutical prescriptions. For co-payments related to hospitalisations, and to account for out-of-pocket limits to the amount the patient can be held accountable for during one calendar year, we assumed patients had no healthcare resource use unrelated to their original ESUS event.

## Results

### Recurrent stroke event rates

Analysis of the CRYSTAL-AF outcomes data showed that ICM patients with a previous ESUS who met enrollment criteria for NAVIGATE ESUS and RE-SPECT ESUS trials and were free of AF after 36 months of monitoring (n = 93) experienced two strokes over a total follow-up of 1890.5 months, resulting in a stroke rate of 1.27 events (95% CI 0.15–4.59) per 100 patient-years. In the base-case model extrapolation, 8.8% of ESUS patients proven free of AF were predicted to have recurrent ischaemic stroke when on aspirin throughout their lifetime. In addition, the model projected that 2.3% of patients would suffer a haemorrhagic stroke, 1.6% other intracranial bleeds, and 87.5% would experience extracranial or CRNM bleeds.

### Clinical efficacy

Table 1 summarises the projected stroke and bleed events for the UK and Additional file 1: Tables S5–S8 show the detailed event projections for the other four examined settings. Based upon the hypothesised 30% reduction in relative risk of recurrent stroke, use of a DOAC was predicted to be associated with a recurrent ischaemic stroke risk of 6.2% per patient. However, the model predicted a substantial increase in bleeding rates; a 20% and 33% higher rate of bleed events projected with dabigatran and rivaroxaban respectively compared with aspirin, accounting for an additional 17.9–29.9 bleeding events per 100 patients. Over a lifetime, the model projected that all dabigatran and rivaroxaban patients would experience at least 1 serious bleed. The combined rate of stroke and bleeding events was 15% and 27% higher with dabigatran and rivaroxaban respectively, compared with aspirin.

**Table 1:** Projected Clinical Events per Patient and over Patient Lifetime in the United Kingdom

Events over patient lifetime per patient		Aspirin	Dabigatran 150 mg	Rivaroxaban 20 mg
**Total ischaemic stroke events**		0.08788	0.06151	0.06151
Δ DOAC vs. Aspirin *(Negative* = *Reduction; Positive* = *Increase)*			− 0.02636	− 0.02636
% DOAC vs. Aspirin *(Negative* = *Reduction; Positive* = *Increase)*			− 30%	− 30%
**Total bleeding events**		0.91371	1.09226	1.21246
Δ DOAC vs. Aspirin			0.17855	0.29875
% DOAC vs. Aspirin			20%	33%
**Haemorrhagic stroke**		0.02292	0.01432	0.02360
Δ DOAC vs. Aspirin			− 0.00859	0.00069
% DOAC vs. Aspirin			− 38%	3%
**Other ICH**		0.01549	0.01007	0.01596
Δ DOAC vs. Aspirin			− 0.00542	0.00046
% DOAC vs. Aspirin			− 35%	3%
**ECH and CRNM bleeds**		0.87530	1.06787	1.17290
Δ DOAC vs. Aspirin			0.19257	0.29760
% DOAC vs. Aspirin			22%	34%
**Total events**	1.00159		1.15377	1.27397
Δ DOAC vs. Aspirin			0.15218	0.27238
% DOAC vs. Aspirin			15%	27%

Table 1 summarises the projected stroke and bleed events for the United Kingdom. Additional file 1: S5–S8 show the detailed event projections for the other four examined settings. Calculation differs by setting due to minor differences in underlying patient survival

### Economic endpoints

Table 2 provides an overview of all costs, by event type, discounted and for every examined healthcare setting. Briefly, DOACs would add between £5953 and £7018 per patient (UK), €6683–€7368 (Netherlands), €4933–€9378 (Spain), AUD$5353–$6539 (Australia) and $26,768–$32,259 (U.S.) from a payer perspective, and were substantially cost-additive under every setting studied, despite the hypothetical 30% relative risk reduction in recurrent stroke. Under a scenario in which DOACs prevent all recurrent strokes, the model suggests that the strategy would remain cost-additive in all countries (data not shown). The impact of baseline stroke risk was also tested. At the upper confidence level (4.59 strokes per 100 patient-years) of the baseline 1.27 stroke risk, DOACs would remain cost-additive in all geographies. Threshold analysis indicated that the lowest ischaemic stroke risk required in order for DOACs to be cost-saving compared to aspirin was 4.80 strokes per 100 patient-years, though the threshold was highly dependent on the geography and choice of DOAC (Additional file 1: Table S9).

**Table 2:** Per-patient Lifetime Costs under the Main Examined Medication Scenarios, by Resource Type and Healthcare Setting

		Aspirin	Dabigatran 150 mg	Rivaroxaban 20 mg	Δ Dabigatran vs. Aspirin	Δ Rivaroxaban vs. Aspirin
United States	Ischaemic Strokes	$8,477.30	$5,934.11	$5,934.11	− $2,543.19	− $2,543.19
	Bleeding Events	$31,867.78	$37,443.42	$41,953.51	$5,575.64	$10,085.73
	Prescriptions	$259.38	$23,994.99	$24,976.10	$23,735.61	$24,716.72
	TOTAL	$40,604.46	$67,372.52	$72,863.72	$26,768.06	$32,259.26
United Kingdom	Ischaemic Strokes	£4,953.06	£3,467.14	£3,467.14	− £1,485.92	− £1,485.92
	Bleeding Events	£2,619.19	£2,565.92	£3,181.65	− £53.27	£562.46
	Prescriptions	£123.19	£7,615.42	£8,064.49	£7,492.23	£7,941.30
	TOTAL	£7,695.44	£13,648.49	£14,713.29	£5,953.04	£7,017.84
Netherlands	Ischaemic Strokes	€1,141.41	€798.99	€798.99	− €342.42	− €342.42
	Bleeding Events	€3,342.24	€3,934.81	€4,404.05	€592.57	€1,061.81
	Prescriptions	€3,173.39	€9,605.93	€9,821.69	€6,432.54	€6,648.30
	TOTAL	€7,657.04	€14,339.73	€15,024.73	€6,682.69	€7,367.69
Spain	Ischaemic Strokes	€2,377.23	€1,664.06	€1,664.06	− €713.17	− €713.17
	Bleeding Events	€4,640.58	€5,299.34	€6,029.50	€658.76	€1,388.92
	Prescriptions	€986.27	€5,974.15	€9,689.00	€4,987.88	€8,702.73
	TOTAL	€8,004.08	€12,937.55	€17,382.57	€4,933.47	€9,378.48
Australia	Ischaemic Strokes	AUD$7,141.27	AUD$4,998.89	AUD$4,998.89	− AUD$2,142.38	− AUD$2,142.38
	Bleeding Events	AUD$6,328.85	AUD$6,831.18	AUD$8,016.72	AUD$502.33	AUD$1,687.87
	Prescriptions	AUD$554.31	AUD$7,547.08	AUD$7,548.11	AUD$6,992.77	AUD$6,993.80
	TOTAL	AUD$14,024.43	AUD$19,377.15	AUD$20,563.72	AUD$5,352.72	AUD$6,539.29

Please note the relative reduction of 30% was assumed equal for dabigatran and rivaroxaban. Note that results would remain cost-additive even if a 100% reduction in ischaemic strokes vs. aspirin was assumed with the DOACs

Table 3 presents the co-pays patients in the U.S., insured by various prevalent commercially available plans, would have to bear. Depending on the level of preference in the formulary of each payer, patients would need to bear $2468.24–$12,844.07 of pharmaceutical cost.

**Table 3:** Patient Out-of-pocket Pharmacy Costs over Lifetime under Various Commercially Available Plans in the U.S

Patient plan	Dabigatran 150 mg (Pradaxa) Patient coinsurance	Rivaroxaban 20 mg (Xarelto) Patient coinsurance
United Healthcare Group Commercial Health Plan (CHP) – coinsurance = 20%	$2468.24	$2568.81
United Healthcare Group Preferred Provider Organization (PPO) Plan – coinsurance = 20%	$2468.24	$2568.81
Anthem High Deductible Health Plan (HDHP) – coinsurance = 20%	$4936.49	$5137.63
Aetna – coinsurance = 20%	$12,341.22	$12,844.07
American Association of Retired Professionals (AARP) Walgreens Plan – coinsurance = 32%	$6773.67	$6783.45
American Association of Retired Professionals (AARP) Preferred Plan – coinsurance = 41%	$5059.90	$5266.07

More information on daily medication costs can be found in Additional file 1: Table S7

The total costs associated with DOACs, including pharmaceutical cost plus any bleeds associated with the safety profile of the drugs, were £7438.96 for Dabigatran 150 mg and £8503.76 for Rivaroxaban, as compared with aspirin (Table 2). In this population of ESUS patients ultimately found to be free of AF, these costs exceed the expense of long-term ICM monitoring for AF tabulated in the CRYSTAL-AF economic evaluation (£3088.61 inclusive of the ICM device, insertion, monitoring, and explant), indicating that a strategy of looking for AF would be economically dominant to an anticoagulate-all approach.

## Exploratory analysis on patients with confirmed AF

While it has been established via the aforementioned CRYSTAL-AF economic analysis [22] that a personalised strategy of oral anticoagulation following a diagnosis of AF made with an ICM was a cost-effective strategy in the cryptogenic stroke/ESUS population, we undertook a threshold analysis to estimate the AF prevalence at which empiric anticoagulation would be economically dominant (that is, superior outcomes at a reduced cost) to a personalised approach. Results indicated that above an AF prevalence of 69.8% or 76.8% (for dabigatran and rivaroxaban, respectively), the empiric anticoagulation approach would be the economically dominant strategy compared to a personalised approach. At this point, the benefits of anticoagulation in the entire population, including those without AF, outweigh the costs of monitoring. On the other hand, in a population in whom all have AF, DOACs were estimated to be cost-saving as the reduction in ischemic stroke risk outweighs the cost of potential adverse events and drug costs associated with DOACs. It should be noted that these analyses were reliant on first determining the risk of stroke recurrence in the CRYSTAL-AF patients meeting ESUS criteria who were indeed confirmed to have AF (n = 28): 0.066 strokes per patient-year (95% CI 0.008–0.240). As the precision around this stroke risk estimate was low due to small sample size, these analyses should be viewed as exploratory in nature.

## Discussion

The objective of this study was to analyse the potential clinical and economic impact of DOAC therapy in ESUS patients proven not to have AF, across multiple international settings. The outcomes of our analyses and simulations show that the use of DOACs in ESUS patients without documented AF may come at an excessive risk of additional bleeds or costs or both, with no clear evidence of providing any benefit. The higher costs associated with additional bleeding events and pharmaceuticals may outweigh possible cost savings associated with reductions in the absolute ischaemic stroke risk, which was found to be orders of magnitude lower than the one observed in the presence of AF. These data provide a potential explanation for the recent NAVIGATE ESUS and RE-SPECT ESUS clinical trial results. The hypotheses driving these studies included that AF was frequently undetected and also that patients without proven AF were at increased risk of (embolic) stroke through other pathways. The former hypothesis was confirmed in CRYSTAL-AF and a recent meta-analysis where one-third of continuously monitored ESUS patients had AF [7, 29].

The second hypothesis driving the concept of anticoagulation for all ESUS patients was the debate on whether AF is the only or main culprit behind CS/ESUS driven by lack of knowledge of the mechanisms underpinning the relationship between AF and stroke [30], and the role of the left atrial appendage (LAA) as a site of thrombus formation [31], especially in CS. Moreover, several other non-AF mechanisms of stroke potentially benefiting from anticoagulation had been proposed including stasis due to an atrial cardiopathy [27] and artery-to-artery embolism [32, 33]

### Pre-DOAC-era studies

Prior to the development of the DOACs, numerous studies had shown that warfarin did not reduce embolic strokes in patients without clinically-evident AF [11–13]. The Warfarin–Aspirin Recurrent Stroke Study (WARSS) in patients with recent non-cardioembolic ischaemic stroke (including 26% with CS) showed no additional benefit over aspirin in preventing recurrent ischaemic stroke or death, while the rate of minor haemorrhagic events was greater with warfarin [11]. A common criticism of WARSS was that the study’s INR target range of 1.4 to 2.8 included INR values below that considered as therapeutic (typically 2.0–3.0). Whilst it does seem to be case that patients outside the 2.0–3.0 therapeutic range gain less benefit in terms of stroke prevention and suffer more bleeds [34], the Warfarin–Aspirin Symptomatic Intracranial Disease (WASID) trial in patients with stroke or TIA due to intracranial atherosclerotic stenosis also reported no benefit of warfarin in preventing ischaemic stroke, or death, but described higher rates of major haemorrhage despite more accepted INR levels [12]. However, the hazard ratio for ischaemic stroke risk in WASID was directionally supportive.

Subsequently, the Warfarin versus Aspirin in Reduced Cardiac Ejection Fraction (WARCEF) trial provided additional data in patients with heart failure in sinus rhythm [13]. Here also, despite no difference between warfarin and aspirin in the composite primary endpoint of ischaemic stroke, intracerebral haemorrhage, or death, there was a 52% lower rate of ischaemic stroke in the warfarin arm, albeit offset by an increased risk of major haemorrhage. Although the Aortic Arch Related Cerebral Hazard (ARCH) trial [35] did not show the same trend, overall, these data hinted at the promise of net clinical benefit in a broad spectrum of patients if bleeding risk could be reduced.

### Post-DOAC-era studies

The promising pre-DOAC-era data led to the development of two large studies using dabigatran and rivaroxaban in patients with ESUS. NAVIGATE ESUS [20], comparing rivaroxaban to aspirin, was halted early for futility due to a higher risk of bleeding in the rivaroxaban arm and no evidence of efficacy in preventing either the primary efficacy outcome of recurrent stroke or systemic embolism, or secondary efficacy outcomes. The increased risk conferred by rivaroxaban (6.5× that of aspirin for haemorrhagic stroke and 2.34× for life-threatening and major bleeding in general) was higher than found in our modelling, suggesting that the ESUS population may be at higher bleeding risk than the stroke-free populations with underlying AF collated by Tawfik et al. [24] Another possible explanation is that because follow-up was incomplete when those findings emerged and the decision was reached to stop the trial, the results may have ultimately been less extreme if more follow-up had accrued. On the other hand, the rates of recurrent ischaemic stroke were identical (4.7%) in the rivaroxaban and aspirin arms. The RE-SPECT ESUS trial [21], comparing dabigatran to aspirin, also found no significant reduction in the primary efficacy endpoint of recurrent stroke which was reduced by 16% (rather than the anticipated 30%), but a significant increase in major and CRNM bleeds in the dabigatran arm.

Our analysis of ESUS patients from CRYSTAL-AF [7] who were free of AF at 3 years (representing approximately two-thirds of the enrolled ESUS population) showed an annual ischaemic stroke risk of 0.0127 per patient-year, very close to that of the aspirin arm of WARCEF (0.0136 per patient per year). Whereas the control arms of WARSS and WASID had rates of 0.08 and 0.207 ischaemic stroke rates respectively, the aspirin arms of NAVIGATE ESUS and RESPECT-ESUS reported 0.047 per year. There is therefore considerable variability in event rates, implying differences between patients that have not yet been fully explored. Overall however, the data from CRYSTAL-AF suggest that the stroke recurrence rate is very low in the absence of AF, such that the opportunity to reduce stroke via anticoagulation may be limited. Our results demonstrate that DOACs would generally be cost-additive and with the very low rate of stroke, combined with the risk of bleeding, this raises uncertainty on the use of these medications for patients without AF, even if clinical efficacy could be shown.

### Personalised care

On the whole, personalised care, if achievable, leads to heightened outcomes compared to imprecise approaches to therapy. Our modelling, the data from NAVIGATE-ESUS and RESPECT-ESUS, and recent meta-analysis [36] and observational data [37] on outcomes in patients with long-term monitoring suggest that this paradigm also holds for anticoagulation in patients with CS. For example, the original CRYSTAL-AF economic analysis found that insertable cardiac monitors are a cost-effective tool to diagnose occult AF and guide appropriate treatment in a CS population [22], with cost-effectiveness further enhanced in subgroups with a slightly higher incidence of AF such as ESUS. While the use of DOACs have been shown to be cost-effective over warfarin and aspirin in a population with proven AF [18, 19], our threshold analysis found that in the ESUS population the rate of AF would have to exceed 69.8% for the empiric anticoagulation strategy be economically dominant (i.e., superior outcomes at a lower cost) compared to a personalised approach. While the rates of underlying AF in the ESUS trials are unknown, existing data show approximately one-third of ESUS patients have AF uncovered after continuous monitoring [7, 29] suggesting that what is currently being proposed as standard of care – i.e., use of long-term cardiac monitoring to guide anticoagulation decisions with NOACs – appears to be rational in this population. In combination with the results of the ESUS trials, these results reinforce the logic of actively looking for AF rather than prescribing DOACs to all patients.

### Limitations

The most important limitation to this analysis is that our findings are based on a small sample size with relatively limited follow-up and rely on linked evidence about benefits and harms. Furthermore, the ESUS criteria were applied retrospectively to the CRYSTAL-AF population [7]. However, the necessary diagnostic information was available to perform this re-classification, as a comprehensive stroke work-up was performed at trial entry.

Due to structural constraints, the model is only able to simulate the risk of up to one recurrent ischaemic stroke event after the original ESUS event. Thus, it is possible that patients could experience multiple ischaemic strokes that could be prevented by anticoagulation, which we have not been able to account for. The influence of this would be to reduce the benefit of the treatment effect of empiric anticoagulation, by underestimating the impact of the hypothetical 30% relative risk reduction. However, with the low observed rate of incident stroke, we expect the actual impact to be small and the direction of our conclusions not to change.

## Conclusion

In a population of patients with unexplained stroke in whom AF has been conclusively excluded through continuous device-based monitoring, our data suggest that empiric anticoagulation in the absence of AF is not clinically or economically useful. Due to the very low underlying rate of recurrent stroke in this patient population, even if DOACs reduced the risk by 30% as proposed in the design of the recent ESUS trials, DOACs would not be cost-saving. An exploratory threshold analysis suggests that the rate of undiagnosed AF would have to be approximately 70% for an empiric ‘anticoagulate-all’ ESUS strategy to offer better outcomes at overall lower cost. These data support the personalised approach of identification of risk factors for stroke (such as AF) and treating these accordingly.

## Supplementary Information

**Additional file 1.** Supplementary information on patient characteristics, model inputs for mortality rates and unit costs for clinical events/pharmaceuticals, and projected clinical event results by geography.

## Data Availability

The datasets used and/or analysed during the current study available from the corresponding author on reasonable request.
