# Supplementary material for: Burden of oral anticoagulation in embolic stroke of undetermined source without atrial fibrillation
Source: BMC Cardiovasc Disord. 2021 Mar 31;21:160. doi: 10.1186/s12872-021-01967-x (PMC8015049; doi:10.1186/s12872-021-01967-x)
Supplement: Supplementary file 1 — Additional file 1. Supplementary information on patient characteristics, model inputs for mortality rates and unit costs for clinical events/pharmaceuticals, and projected clinical event results by geography. [file 12872_2021_1967_MOESM1_ESM.docx]

**Supplementary Files**

**Burden of Oral Anticoagulation in Embolic Stroke of** **Undetermined Source without Atrial Fibrillation**

**Table of Contents**

[Supplementary Table 1. Patient Characteristics at Baseline in CRYSTAL-AF ESUS Patients (ICM Cohort) 2](#_Toc59172119)

[Supplementary Table 2. Non-cerebrovascular Mortality – by Geography 4](#_Toc59172120)

[Supplementary Table 3. Unit Costs for Stroke and Bleed-related Events 8](#_Toc59172121)

[Supplementary Table 4. Daily Medication Costs 10](#_Toc59172122)

[Supplementary Table 5. Projected Clinical Events per Patient and over Patient Lifetime with the Main Examined Prescription Scenarios – United States 11](#_Toc59172123)

[Supplementary Table 6. Projected Clinical Events per Patient and over Patient Lifetime with the Main Examined Prescription Combinations – The Netherlands 12](#_Toc59172124)

[Supplementary Table 7. Projected Clinical Events per Patient and over Patient Lifetime with the Main Examined Prescription Combinations – Spain 13](#_Toc59172125)

[Supplementary Table 8. Projected Clinical Events per Patient and over Patient Lifetime with the Main Examined Prescription Combinations – Australia 14](#_Toc59172126)

[Supplementary Table 9. Threshold Analysis - Baseline Ischemic Stroke Risks Required for NOACs to be Cost-saving Versus Aspirin 15](#_Toc59172127)

Supplementary Table 1. Patient Characteristics at Baseline in CRYSTAL-AF ESUS Patients (ICM Cohort)

| **Subject Characteristics** | **ESUS with AF (N = 28)** | **ESUS Non-AF (N = 93)** |
| --- | --- | --- |
| **Age (Years**) | | |
| Mean ± Standard Deviation | 67.1 ± 10.0 | 59.6 ± 11.1 |
| Median | 66.0 | 61.0 |
| 25^th^ Percentile - 75^th^ Percentile | 60.5 - 76.0 | 49.0 - 68.0 |
| Minimum - Maximum | 49.0 - 87.0 | 40.0 - 80.0 |
| **Sex - n (%)** | | |
| Male | 20 (71.4%) | 54 (58.1%) |
| Female | 8 (28.6%) | 39 (41.9%) |
| **Index Event Type - n (%)** | | |
| Stroke | 25 (89.3%) | 84 (90.3%) |
| TIA | 3 (10.7%) | 9 (9.7%) |
| **CHADS_2_ Score - n (%)** | | |
| 2 | 7 (25.0%) | 35 (37.6%) |
| 3 | 13 (46.4%) | 46 (49.5%) |
| 4 | 8 (28.6%) | 10 (10.8%) |
| 5 | 0 (0.0%) | 2 (2.2%) |
| 6 | 0 (0.0%) | 0 (0.0%) |
| **CHADS_2_ Score** | | |
| Mean ± Standard Deviation | 3.0 ± 0.7 | 2.8 ± 0.7 |
| Median | 3.0 | 3.0 |
| 25^th^ Percentile - 75^th^ Percentile | 2.5 - 4.0 | 2.0 - 3.0 |
| Minimum - Maximum | 2.0 - 4.0 | 2.0 - 5.0 |
| **Modified Rankin Scale - n (%)** | | |
| 0-2 | 26 (92.9%) | 89 (95.7%) |
| >2 | 2 (7.1%) | 4 (4.3%) |
| **NIH Stroke Score** | | |
| Mean ± Standard Deviation | 1.2 ± 1.5 | 0.7 ± 1.3 |
| Median | 1.0 | 0.0 |
| 25^th^ Percentile - 75^th^ Percentile | 0.0 - 2.0 | 0.0 - 1.0 |
| Minimum - Maximum | 0.0 - 6.0 | 0.0 - 10.0 |

Age-dependent baseline mortality in the model was based on rates from geography-specific life tables. Since cerebrovascular events and their associated risks were explicitly modeled, background mortality rates were adjusted to exclude deaths due to cerebrovascular events, as described in Table 2 below.

Note: following a secondary non-fatal stroke, an individual’s mortality risk increased depending on the severity of the stroke and their treatment, based on the framework described in the aforementioned economic framework (Diamantopoulos A, Sawyer LM, Lip G et al. International Journal of Stroke 2016, Vol. 11(3) 302–312). Briefly, the mortality hazard ratios associated with a secondary mild, moderate, and severe stroke were 2.56, 4.63, and 13.19, respectively.

Supplementary Table 2. Non-cerebrovascular Mortality – by Geography

| **United Kingdom & The Netherlands^1^** | | | | | | |
| --- | --- | --- | --- | --- | --- | --- |
|  | **All-cause Mortality** | | **CVD Deaths**  **(ICD10 I60-I69)** | | **Cerebrovascular deaths as % of all deaths** | |
| **Age** | **Male** | **Female** | **Male** | **Female** | **Male** | **Female** |
| 40-44 years | 3,360 | 2,043 | 111 | 66 | 3.3% | 3.2% |
| 45-49 years | 4,962 | 3,363 | 183 | 118 | 3.7% | 3.5% |
| 50-54 years | 6,697 | 4,705 | 235 | 209 | 3.5% | 4.4% |
| 55-59 years | 9,404 | 6,633 | 310 | 310 | 3.3% | 4.7% |
| 60-64 years | 14,889 | 10,212 | 479 | 413 | 3.2% | 4.0% |
| 65-69 years | 21,054 | 14,609 | 827 | 673 | 3.9% | 4.6% |
| 70-74 years | 25,892 | 18,874 | 1,274 | 1,079 | 4.9% | 5.7% |
| 75-79 years | 34,120 | 28,050 | 2,051 | 2,138 | 6.0% | 7.6% |
| 80-84 years | 41,889 | 42,662 | 2,904 | 3,909 | 6.9% | 9.2% |
| 85-89 years | 39,335 | 53,117 | 3,147 | 5,463 | 8.0% | 10.3% |
| 90-94 years | 23,064 | 45,702 | 1,889 | 4,854 | 8.2% | 10.6% |
| 95-99 years | 7,265 | 24,256 | 591 | 2,402 | 8.1% | 9.9% |
| **United States^2^** | | | | | | |
|  | **All-cause Mortality** | | **CVD Deaths**  **(ICD10 I60-I69)** | | **Cerebrovascular deaths as % of all deaths** | |
| **Age** | **Male** | **Female** | **Male** | **Female** | **Male** | **Female** |
| 40-44 years | 26,406 | 16,109 | 662 | 490 | 2.5% | 3.0% |
| 45-49 years | 39,769 | 25,816 | 1,087 | 902 | 2.7% | 3.5% |
| 50-54 years | 65,317 | 42,614 | 1,891 | 1,473 | 2.9% | 3.5% |
| 55-59 years | 98,650 | 63,496 | 2,981 | 2,210 | 3.0% | 3.5% |
| 60-64 years | 123,734 | 80,565 | 4,179 | 2,940 | 3.4% | 3.6% |
| 65-69 years | 144,389 | 102,266 | 5,293 | 4,344 | 3.7% | 4.2% |
| 70-74 years | 148,353 | 117,072 | 6,292 | 5,839 | 4.2% | 5.0% |
| 75-79 years | 156,851 | 138,044 | 7,730 | 8,236 | 4.9% | 6.0% |
| 80-84 years | 167,968 | 174,053 | 9,378 | 12,445 | 5.6% | 7.2% |
| 85-89 years | 168,063 | 216,416 | 9,628 | 17,384 | 5.7% | 8.0% |
| 90-94 years | 113,337 | 197,434 | 6,695 | 16,355 | 5.9% | 8.3% |
| 95-99 years | 36,909 | 94,467 | 2,268 | 7,762 | 6.1% | 8.2% |
| **Spain^3^** | | | | | | |
|  | **All-cause Mortality** | | **CVD Deaths**  **(ICD10 I60-I69)** | | **Cerebrovascular deaths as % of all deaths** | |
| **Age** | **Male** | **Female** | **Male** | **Female** | **Male** | **Female** |
| 40-44 years | 2,296 | 1,272 | 69 | 40 | 3.0% | 3.1% |
| 45-49 years | 4,021 | 2,128 | 142 | 76 | 3.5% | 3.6% |
| 50-54 years | 6,860 | 3,422 | 234 | 112 | 3.4% | 3.3% |
| 55-59 years | 9,639 | 4,545 | 358 | 184 | 3.7% | 4.0% |
| 60-64 years | 12,508 | 5,522 | 419 | 207 | 3.3% | 3.7% |
| 65-69 years | 16,602 | 7,536 | 664 | 350 | 4.0% | 4.6% |
| 70-74 years | 20,905 | 10,705 | 1,026 | 643 | 4.9% | 6.0% |
| 75-79 years | 25,749 | 16,783 | 1,505 | 1,259 | 5.8% | 7.5% |
| 80-84 years | 37,894 | 33,998 | 2,506 | 2,959 | 6.6% | 8.7% |
| 85-89 years | 37,901 | 48,674 | 2,501 | 4,291 | 6.6% | 8.8% |
| 90-94 years | 23,225 | 43,207 | 1,625 | 3,706 | 7.0% | 8.6% |
| 95-99 years | 7,041 | 21,444 | 437 | 1,687 | 6.2% | 7.9% |
| **Australia^4^** | | | | | | |
|  | **All-cause Mortality** | | **CVD Deaths**  **(ICD10 I60-I69)** | | **Cerebrovascular deaths as % of all deaths** | |
| **Age** | **Male** | **Female** | **Male** | **Female** | **Male** | **Female** |
| 40-44 years | 2,186 | 1,256 | 37 | 47 | 1.7% | 3.7% |
| 45-49 years | 4,310 | 2,722 | 134 | 112 | 3.1% | 4.1% |
| 50-54 years | 4,310 | 2,722 | 134 | 112 | 3.1% | 4.1% |
| 55-59 years | 8,532 | 5,255 | 242 | 184 | 2.8% | 3.5% |
| 60-64 years | 8,532 | 5,255 | 242 | 184 | 2.8% | 3.5% |
| 65-69 years | 15,175 | 9,542 | 633 | 411 | 4.2% | 4.3% |
| 70-74 years | 15,175 | 9,542 | 633 | 411 | 4.2% | 4.3% |
| 75-79 years | 22,765 | 18,023 | 1,353 | 1,456 | 5.9% | 8.1% |
| 80-84 years | 22,765 | 18,023 | 1,353 | 1,456 | 5.9% | 8.1% |
| 85-89 years | 22,878 | 30,262 | 1,609 | 3,122 | 7.0% | 10.3% |
| 90-94 years | 22,878 | 30,262 | 1,609 | 3,122 | 7.0% | 10.3% |
| 95-99 years | 3,065 | 7,983 | 221 | 864 | 7.2% | 10.8% |

^1^Office for National Statistics. Deaths registered in England and Wales (series DR): 2012. Available at: http://www.ons.gov.uk/ons/rel/vsob1/mortality-statistics--deaths-registered-in-england-and-wales--series-dr-/2012/index.htm. Accessed February 24, 2018.

^2^U.S. Center for Disease Control. CDC Wonder. Cardiovascular Deaths (Chapter I of ICD-10 Codes) grouped by 5-year groups. Available at: https://wonder.cdc.gov/ucd-icd10.html. Accessed February 24, 2018.

^3^Spanish Office of Statistics. INEBase Available at: http://www.ine.es/jaxi/Datos.htm?path=/t15/p417/a2016/l1/&file=01000.px. Accessed March 2, 2018.

^4^Australian Bureau of Statistics. 3303.0 – Causes of Death, Australia 2018. Available at: http://www.abs.gov.au/ausstats/abs@.nsf/0/97B8700384EBDC9BCA2576E800154AEB?Opendocument. Accessed February 25, 2018.

Supplementary Table 3. Unit Costs for Stroke and Bleed-related Events

| **Description of Events** | **Cost** | | | | |
| --- | --- | --- | --- | --- | --- |
|  | **United Kingdom^1-2^** | **United States^3-4^** | **The Netherlands^5^** | **Spain^6-7^** | **Australia^8^** |
| **Acute stroke event costs** | | | | | |
| Mild IS | £3,401.00 | $49,764.28 | € 2,275.00 | 6,256.65 € | AUD $23,297.50 |
| Moderate IS | £17,743.00 | $49,764.28 | € 5,575.00 | 6,582.89 € | AUD $25,671.00 |
| Severe IS | £24,234.00 | $49,764.28 | € 17,831.00 | 7,284.66 € | AUD $22,938.00 |
| Fatal IS | £3,059.00 | $49,764.28 | € 2,275.00 | 6,193.76 € | AUD $16,624.00 |
| Mild HS | £9,903.00 | $72,770.03 | € 2,166.00 | 7,939.06 € | AUD $23,297.50 |
| Moderate HS | £25,442.00 | $72,770.03 | € 6,374.00 | 8,265.29 € | AUD $25,671.00 |
| Severe HS | £43,036.00 | $72,770.03 | € 18,881.00 | 8,967.07 € | AUD $22,938.00 |
| Fatal HS | £1,592.00 | $72,770.03 | € 2,166.00 | 7,876.18 € | AUD $16,624.00 |
| **Other event costs** | | | | | |
| Non-Fatal Other ICH | £4,007.72 | $72,770.03 | €9,140.00 | €8,167.20 | AUD $11,194.91 |
| Fatal Other ICH | £4,007.72 | $72,770.03 | €9,140.00 | €8,167.20 | AUD $11,194.91 |
| Non-Fatal ECH | £5,798.55 | $38,748.16 | €1,522.00 | €3,732.12 | AUD $4,065.70 |
| Fatal ECH | £1,964.00 | $38,748.16 | €1,522.00 | €8,167.20 | AUD $4,065.70 |
| CRNM Bleed | £412.37 | $14,129.14 | €614.00 | €2,415.28 | AUD $2,609.55 |
| **Post-stroke (IS or HS) 1-year costs** | | | | | |
| Post Mild Stroke | £65,615.22 | $83,455.88 | € 12,681.25 | 12,707.90 € | AUD $106,735.63 |
| Post Moderate Stroke | £83,870.70 | $86,353.51 | € 20,040.68 | 53,347.22 € | AUD $103,482.82 |
| Post Severe Stroke | £46,086.14 | $113,965.44 | € 10,017.12 | 59,437.53 € | AUD $57,283.54 |

Costs represent payer perspective costs and have been inflated to 2017 dollars.

Abbreviations: CRNM, clinically relevant non-major bleed; ECH, extracerebral hemorrhage; HS, hemorrhagic stroke; ICH, intracerebral hemorrhage; IS, ischaemic stroke.

^1^Luengo-Fernandez R, Paul NL, Gray AM, et al. Population-based study of disability and institutionalization after transient ischemic attack and stroke: 10-year results of the Oxford Vascular Study. Stroke 2013; 44: 2854–2861.

^2^National Health Service (NHS) Digital. Acute Patient Level Activity and Costing - Data Quality and Analysis of Expanded Pilot, 2016-17. Published July 19, 2018. Available at: https://digital.nhs.uk/data-and-information/publications/statistical/mi-acute-patient-level-activity-and-costing/data-quality-and-initial-analysis-2016-17.

^3^Shireman TI, Wang K, Saver JL, et al. Cost-Effectiveness of Solitaire Stent Retriever Thrombectomy for Acute Ischemic Stroke: Results From the SWIFT-PRIME Trial. Stroke. 2017; 48:379-387.

^4^Medtronic data on file. Analysis of Instant Health Data database (Boston Health Economics, Boston, MA, USA) for calendar years 2015-2016. Accessed November 22, 2018.

^5^The Dutch Healthcare Authority (Nederlandse Zorgautoritait – NZa). Open data from the Dutch Healthcare Authority, 2017. Available at: www.opendisdata.nl.

^6^Baron Esquivias G, Escolar Albaladejo G, Zamorano JL, Betegon Nicolas L, Canal Fontcuberta C, de Salas-Cansado M, et al. Cost-effectiveness Analysis Comparing Apixaban and Acenocoumarol in the Prevention of Stroke in Patients With Nonvalvular Atrial Fibrillation in Spain. Rev Esp Cardiol (Engl Ed) 2015;68(8):680-90.

^7^2017 data of the Health Information Institute of the Ministry of Health and Social Services. Instituto de Información Sanitaria; Ministerio de Sanidad, Servicios Sociales e Igualdad.

^8^Australian Medical Services Advisory Committee (MSAC). Public Summary Document: Implantable loop recorders for diagnosis of atrial fibrillation in cryptogenic stroke. Published April 7, 2017. Available at: http://www.msac.gov.au/internet/msac/publishing.nsf/Content/1443-public.

Supplementary Table 4. Daily Medication Costs

| **Medication** | **Cost per Day (2017 Dollars)** | | | | |
| --- | --- | --- | --- | --- | --- |
|  | **United Kingdom^1^** | **United States^2^** | **The Netherlands^3^** | **Spain^4^** | **Australia^5^** |
| **ASA (Aspirin)** | £0.03 | $0.06 | €0.74 | €0.20 | AUD $0.13 |
| **Dabigatran 150mg** | £1.70 | $5.55 | €2.24 | €1.21 | AUD $1.77 |
| **Rivaroxaban** | £1.80 | $5.78 | €2.29 | €1.96 | AUD $1.77 |

^1^Royal Pharmaceutical Society. British National Formulary (BNF). Available at: https://www.medicinescomplete.com/mc/bnf/current/.

^2^Medtronic data on file. Analysis of Instant Health Data database (Boston Health Economics, Boston, MA, USA) for calendar years 2015-2016. Accessed November 22, 2018.

^3^The Netherlands Costing Institute (Zorginstituut Nederland). Pharmacy purchase price database. Available at: https://www.medicijnkosten.nl/.

^4^2017 data of the Health Information Institute of the Ministry of Health and Social Services. Instituto de Información Sanitaria; Ministerio de Sanidad, Servicios Sociales e Igualdad.

^5^Australian Medical Services Advisory Committee (MSAC). Public Summary Document: Implantable loop recorders for diagnosis of atrial fibrillation in cryptogenic stroke. Published April 7, 2017. Available at: http://www.msac.gov.au/internet/msac/publishing.nsf/Content/1443-public.

Supplementary Tables 5-8 below summarise the projected stroke and bleed events for the United States, Netherlands, Spain, and Australia. Event rates for the United Kingdom are provided in Table 1 of the article. Calculation differs by setting fur to minor differences in underlying patient survival.

Supplementary Table 5. Projected Clinical Events per Patient and over Patient Lifetime with the Main Examined Prescription Scenarios – United States

| **Events over Patient Lifetime per Patient** | **Aspirin** |  | **Dabigatran 150 mg** | **Rivaroxaban 20 mg** |
| --- | --- | --- | --- | --- |
|  |  |  |  |  |
| **Total Ischaemic Stroke** | 0.08555 |  | 0.05989 | 0.05989 |
| Δ NOAC vs Aspirin (Negative=Reduction; Positive=Increase) | |  | -0.02567 | -0.02567 |
| % NOAC vs Aspirin (Negative=Reduction; Positive=Increase) | |  | -30% | -30% |
|  |  |  |  |  |
| **Total Haemorrhagic Stroke** | 0.02231 |  | 0.01394 | 0.02298 |
| Δ NOAC vs Aspirin (Negative=Reduction; Positive=Increase) | |  | -0.00837 | 0.00067 |
| % NOAC vs Aspirin (Negative=Reduction; Positive=Increase) | |  | -38% | 3% |
|  |  |  |  |  |
| **Total Other ICH** | 0.01508 |  | 0.00980 | 0.01553 |
| Δ NOAC vs Aspirin (Negative=Reduction; Positive=Increase) | |  | -0.00528 | 0.00045 |
| % NOAC vs Aspirin (Negative=Reduction; Positive=Increase) | |  | -35% | 3% |
|  |  |  |  |  |
| **ECH and CRNM Bleeds** | 0.84755 |  | 1.03400 | 1.13571 |
| Δ NOAC vs Aspirin (Negative=Reduction; Positive=Increase) | |  | 0.18646 | 0.28817 |
| % NOAC vs Aspirin (Negative=Reduction; Positive=Increase) | |  | 22% | 34% |

Supplementary Table 6. Projected Clinical Events per Patient and over Patient Lifetime with the Main Examined Prescription Combinations – The Netherlands

| **Events over Patient Lifetime per Patient** | **Aspirin** |  | **Dabigatran 150 mg** | **Rivaroxaban 20 mg** |
| --- | --- | --- | --- | --- |
|  |  |  |  |  |
| **Total Ischaemic Stroke** | 0.08792 |  | 0.06154 | 0.06154 |
| Δ NOAC vs Aspirin (Negative=Reduction; Positive=Increase) | |  | -0.02637 | -0.02637 |
| % NOAC vs Aspirin (Negative=Reduction; Positive=Increase) | |  | -30% | -30% |
|  |  |  |  |  |
| **Total Haemorrhagic Stroke** | 0.02293 |  | 0.01433 | 0.02361 |
| Δ NOAC vs Aspirin (Negative=Reduction; Positive=Increase) | |  | -0.00860 | 0.00069 |
| % NOAC vs Aspirin (Negative=Reduction; Positive=Increase) | |  | -38% | 3% |
|  |  |  |  |  |
| **Total Other ICH** | 0.01550 |  | 0.01007 | 0.01596 |
| Δ NOAC vs Aspirin (Negative=Reduction; Positive=Increase) | |  | -0.00542 | 0.00046 |
| % NOAC vs Aspirin (Negative=Reduction; Positive=Increase) | |  | -35% | 3% |
|  |  |  |  |  |
| **ECH and CRNM Bleeds** | 0.87477 |  | 1.06722 | 1.17220 |
| Δ NOAC vs Aspirin (Negative=Reduction; Positive=Increase) | |  | 0.19245 | 0.29742 |
| % NOAC vs Aspirin (Negative=Reduction; Positive=Increase) | |  | 22% | 34% |

Supplementary Table 7. Projected Clinical Events per Patient and over Patient Lifetime with the Main Examined Prescription Combinations – Spain

| **Events over Patient Lifetime per Patient** | **Aspirin** |  | **Dabigatran 150 mg** | **Rivaroxaban 20 mg** |
| --- | --- | --- | --- | --- |
|  |  |  |  |  |
| **Total Ischaemic Stroke** | 0.09009 |  | 0.06307 | 0.06307 |
| Δ NOAC vs Aspirin (Negative=Reduction; Positive=Increase) | |  | -0.02703 | -0.02703 |
| % NOAC vs Aspirin (Negative=Reduction; Positive=Increase) | |  | -30% | -30% |
|  |  |  |  |  |
| **Total Haemorrhagic Stroke** | 0.02349 |  | 0.01468 | 0.02420 |
| Δ NOAC vs Aspirin (Negative=Reduction; Positive=Increase) | |  | -0.00881 | 0.00070 |
| % NOAC vs Aspirin (Negative=Reduction; Positive=Increase) | |  | -38% | 3% |
|  |  |  |  |  |
| **Total Other ICH** | 0.01588 |  | 0.01032 | 0.01636 |
| Δ NOAC vs Aspirin (Negative=Reduction; Positive=Increase) | |  | -0.00556 | 0.00048 |
| % NOAC vs Aspirin (Negative=Reduction; Positive=Increase) | |  | -35% | 3% |
|  |  |  |  |  |
| **ECH and CRNM Bleeds** | 0.91083 |  | 1.11122 | 1.22052 |
| Δ NOAC vs Aspirin (Negative=Reduction; Positive=Increase) | |  | 0.20038 | 0.30968 |
| % NOAC vs Aspirin (Negative=Reduction; Positive=Increase) | |  | 22% | 34% |

Supplementary Table 8. Projected Clinical Events per Patient and over Patient Lifetime with the Main Examined Prescription Combinations – Australia

| **Events over Patient Lifetime per Patient** | **Aspirin** |  | **Dabigatran 150 mg** | **Rivaroxaban 20 mg** |
| --- | --- | --- | --- | --- |
|  |  |  |  |  |
| **Total Ischaemic Stroke** | 0.09182 |  | 0.06427 | 0.06427 |
| Δ NOAC vs Aspirin (Negative=Reduction; Positive=Increase) | |  | -0.02755 | -0.02755 |
| % NOAC vs Aspirin (Negative=Reduction; Positive=Increase) | |  | -30% | -30% |
|  |  |  |  |  |
| **Total Haemorrhagic Stroke** | 0.02394 |  | 0.01496 | 0.02466 |
| Δ NOAC vs Aspirin (Negative=Reduction; Positive=Increase) | |  | -0.00898 | 0.00072 |
| % NOAC vs Aspirin (Negative=Reduction; Positive=Increase) | |  | -38% | 3% |
|  |  |  |  |  |
| **Total Other ICH** | 0.01619 |  | 0.01052 | 0.01667 |
| Δ NOAC vs Aspirin (Negative=Reduction; Positive=Increase) | |  | -0.00566 | 0.00049 |
| % NOAC vs Aspirin (Negative=Reduction; Positive=Increase) | |  | -35% | 3% |
|  |  |  |  |  |
| **ECH and CRNM Bleeds** | 0.93615 |  | 1.14211 | 1.25444 |
| Δ NOAC vs Aspirin (Negative=Reduction; Positive=Increase) | |  | 0.20595 | 0.31829 |
| % NOAC vs Aspirin (Negative=Reduction; Positive=Increase) | |  | 22% | 34% |

Supplementary Table 9. Threshold Analysis - Baseline Ischemic Stroke Risks Required for NOACs to be Cost-saving Versus Aspirin

| **Minimum Baseline Stroke Risk**  **(Strokes per Patient-year)** | **Dabigatran**  **150 mg** | **Rivaroxaban**  **20 mg** |
| --- | --- | --- |
| **United Kingdom** | 0.074 | 0.085 |
| **United States** | 0.209 | 0.254 |
| **The Netherlands** | 0.960 | 1.000 |
| **Spain** | 0.131 | 0.358 |
| **Australia** | 0.048 | 0.056 |

The impact of varying baseline ischemic stroke risk was tested; thresholds presented in Supplementary Table 9 indicate the minimum stroke risk (rate of strokes per patient-year) at which dabigatran or rivaroxaban were projected to be cost-saving over a lifetime horizon, compared with aspirin. Note: the stroke risk observed in the CRYSTAL AF population of ESUS patients without AF was 0.0127 strokes per patient year (95% CI = 0.0015 - 0.0459).
